# Elicitation of domain knowledge for a machine learning model for paediatric critical illness in South Africa

**DOI:** 10.3389/fped.2023.1005579

**Published:** 2023-02-21

**Authors:** Michael A. Pienaar, Joseph B. Sempa, Nicolaas Luwes, Elizabeth C. George, Stephen C. Brown

**Affiliations:** ^1^ Department of Paediatrics and Child Health, Paediatric Critical Care Unit, University of the Free State, Bloemfontein, South Africa; ^2^ Department of Biostatistics, Faculty of Health Sciences, University of the Free State, Bloemfontein, South Africa; ^3^ Department of Electrical, Electronic and Computer Engineering, Faculty of Engineering, Built Environment and Information Technology, Central University of Technology, Bloemfontein, South Africa; ^4^ Medical Research Council Clinical Trials Unit, University College London, London, United Kingdom; ^5^ Paediatric Cardiology Unit, Department of Paediatrics and Child Health, University of the Free State, Bloemfontein, South Africa

**Keywords:** machine learning, critical care, children, domain knowledge, triage, severity of illness

## Abstract

**Objectives:**

Delays in identification, resuscitation and referral have been identified as a preventable cause of avoidable severity of illness and mortality in South African children. To address this problem, a machine learning model to predict a compound outcome of death prior to discharge from hospital and/or admission to the PICU was developed. A key aspect of developing machine learning models is the integration of human knowledge in their development. The objective of this study is to describe how this domain knowledge was elicited, including the use of a documented literature search and Delphi procedure.

**Design:**

A prospective mixed methodology development study was conducted that included qualitative aspects in the elicitation of domain knowledge, together with descriptive and analytical quantitative and machine learning methodologies.

**Setting:**

A single centre tertiary hospital providing acute paediatric services.

**Participants:**

Three paediatric intensivists, six specialist paediatricians and three specialist anaesthesiologists.

**Interventions:**

None.

**Measurements and main results:**

The literature search identified 154 full-text articles reporting risk factors for mortality in hospitalised children. These factors were most commonly features of specific organ dysfunction. 89 of these publications studied children in lower- and middle-income countries. The Delphi procedure included 12 expert participants and was conducted over 3 rounds. Respondents identified a need to achieve a compromise between model performance, comprehensiveness and veracity and practicality of use. Participants achieved consensus on a range of clinical features associated with severe illness in children. No special investigations were considered for inclusion in the model except point-of-care capillary blood glucose testing. The results were integrated by the researcher and a final list of features was compiled.

**Conclusion:**

The elicitation of domain knowledge is important in effective machine learning applications. The documentation of this process enhances rigour in such models and should be reported in publications. A documented literature search, Delphi procedure and the integration of the domain knowledge of the researchers contributed to problem specification and selection of features prior to feature engineering, pre-processing and model development.

## Introduction

Failures in acute care and critical care systems such as triage, identification and resuscitation present a significant challenge in the treatment of life-threatening illness and injury in low to middle income countries (1). One of the key weaknesses is the failure of triage and identification systems to detect children in need of life-saving care (2). In South Africa, it has been found that failures in identification, accurate assessment of severity of illness, early resuscitation, and timely referral to higher levels of care were responsible for significant avoidable severity of illness and mortality (3).

Machine learning has drawn considerable interest in recent medical literature. Machine learning offers a wide range of possible use-cases in the clinical setting, including diagnosis, prognosis, workflow and improving patient access to services (4). The exploration of these applications has extended to paediatric research (5). Lonsdale et al. and Rajkomar et al., however, both point out that this area is under-explored to date (4, 5). This new field demands a broad interdisciplinary collaboration. In the South African and developing context, it is also vitally important that research be conducted in this field to ensure that capacity is developed to investigate and implement such technology in a manner suited to the unique needs and contexts of our setting.

The integration of human knowledge is a crucial aspect of the development, architecture, interpretation, and use of machine learning models. This includes the integration of human knowledge in a field (domain knowledge) as well as knowledge of learning, the human brain, computer science and statistics (general knowledge) (6). Kerrigan et al. have conducted a survey of the elicitation of domain knowledge in applied machine learning and pointed to the need for the documentation of the elicitation process (7). In doing so, they have developed a taxonomy for the elicitation process that includes the *elicitation goal*, the *elicitation target*, the *elicitation process* and the *use of elicited knowledge*. This has aimed to address the specific challenge of this elicitation process being undertaken in an *ad-hoc* manner (8).

The use of machine learning in triage and mortality prediction has been described in high-income countries. Goto et al. have described the use of machine learning to predict paediatric intensive care unit (PICU) admission and hospitalisation (9). Aczon et al. and Kim et al. have described models for the prediction of mortality in PICU. These models represent significant progress on existing models in their ability to make dynamic or continuous assessments of mortality risk over time (10, 11). There is a paucity of literature describing such applications in (12) Africa. This paucity together with the contribution of failures in triage and identification of critically ill children provide a strong rationale for conducting research in this area and developing clinically implementable tools for the identification of critically ill children that are appropriate to the South African setting. With this rationale, this study undertook to elicit expert clinical domain knowledge to clearly define the goals of and select viable features for predictive models for paediatric critical illness. The application of this work is described in a recent publication by our group in which we describe the prospective development of machine learning models for the identification of critically ill children (12).

## Methods and study design

**Figure 1:**
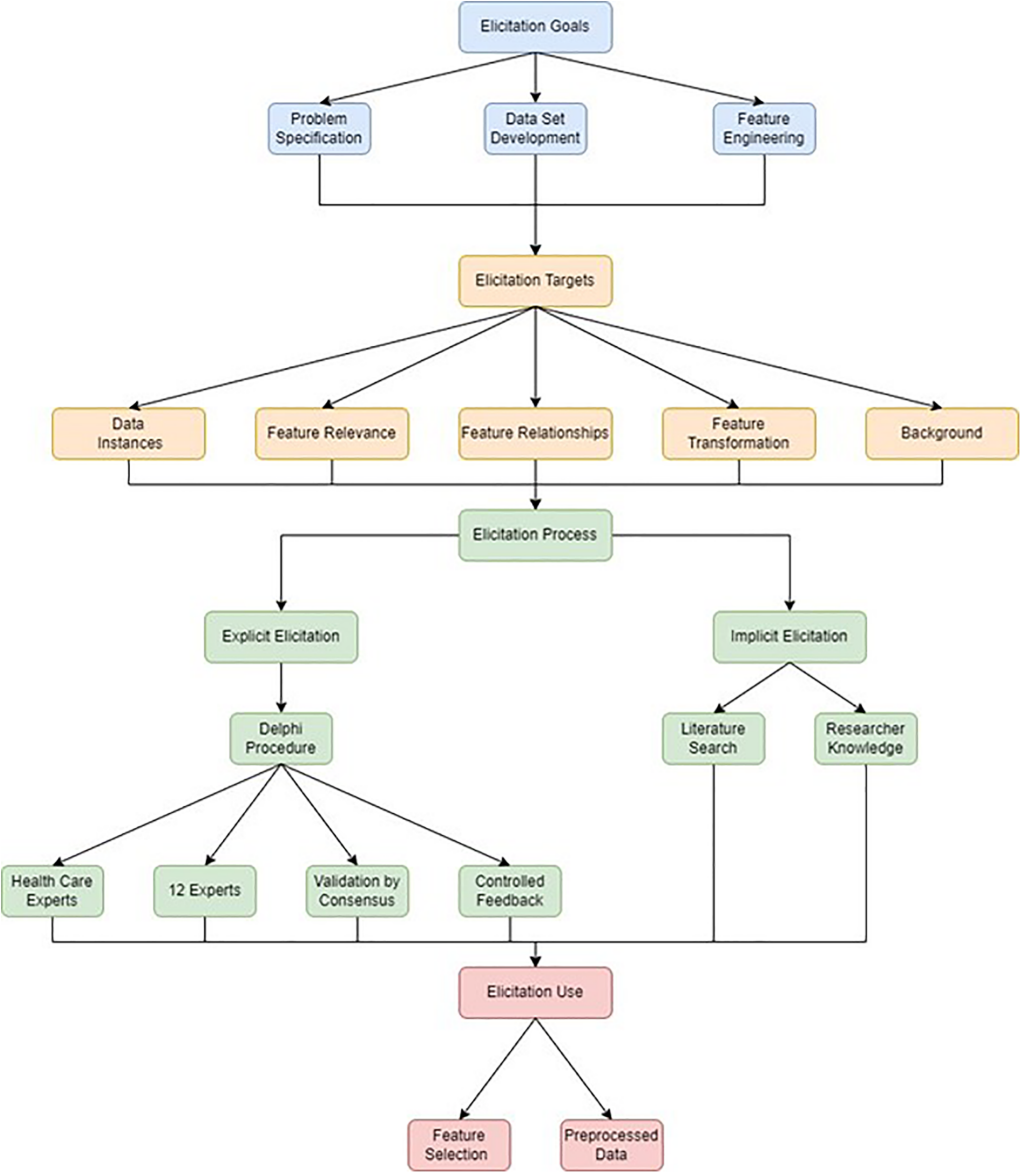
Data elicitation methodology. Data elicitation goals are depicted in blue, data elicitation targets are depicted in orange, data elicitation processes are depicted in green and data elicitation uses are depicted in red.

In this study, the domain knowledge elicitation was documented within the taxonomy proposed by Kerrigan et al. and its role in the various aspects of the development of the machine learning application was described. A literature search was combined with a Delphi procedure to provide a documented account of the elicitation process. By searching the literature, a framework can be developed by which knowledge of a domain can be integrated into further analysis (13). Delphi procedure is a methodology employing both quantitative and qualitative elements to gain consensus of expert opinion using iterative questionnaires (rounds) and controlled feedback (14, 15). The aim of producing a rigorous, documented, reproducible approach to domain knowledge elicitation was pursued by documenting these two processes, their integration and their application at different stages of the model development process. In this study, the data elicitation process employed in problem specification and selection of features is described, but human domain knowledge was also employed in feature engineering and pre-processing steps employed in model development. The overall methodological process employed is summarised in Figure 1.

### Ethical clearance

Internal review board approval was obtained from the Health Sciences Research Ethics Committee (UFS-HSD2020/2204/2505-0003) and the Free State Department of Health (FS_202102_019). Informed consent was obtained in writing from the participants in the Delphi procedure. All data was collected anonymously directly into a REDCap® database and stored on a secure server prior to exporting as a CSV file for analysis.

### Literature search

The literature search was performed using the method described by vom Brocke et al. (13). A Medline database search was conducted, and search terms and results were documented. Articles that reported risk factors for mortality in hospitalised children after 2000 were included. No laboratory values were included. Articles which were not available as full text or in English were excluded. Titles, abstracts, and full text articles were reviewed respectively. Articles were separated into articles from lower- and middle-income countries (LMIC) and high-income countries. A concept matrix was constructed, and the results were summarised.

### Delphi procedure

The Delphi procedure included twelve experts from South Africa. This group was made up of three paediatric intensivists (from the University of the Free State, the University of the Witwatersrand and the private sector respectively), six specialist paediatricians (five from the University of the Free State and one from the private sector) and three specialist anaesthesiologists from the University of the Free State. All participants were anonymous from one another.

The purpose of the Delphi procedure was to set operational priorities for the model relative to the problem specification and to identify candidate features (independent variables) that are likely to be associated with the study outcome. Prior to the start of the Delphi, participants were provided with a summary of the proposed research as well as a table depicting eligible variables included in existing models.

The number of rounds was set *a priori* at three, based on the recommendation of Trevelyan and Robinson (14), feedback from Rounds 2 and 3 was provided as interquartile ranges and medians and consensus (used as an assessment, not an endpoint) was determined by percentage agreement. Consensus was defined as 75% agreement. For Rounds 2 and 3 a five-point Likert scale was employed (strongly disagree, disagree, no comment, agree, strongly agree). All three rounds of the Delphi were conducted using individual online REDCap® surveys.

In Round 1, the participants were asked the following open-ended questions:

1. What are the important characteristics of a variable for inclusion in a study designed to predict severe illness in children in a resource-limited setting?
2. Should completeness or practicality of data collection be given priority in data collection for this study?
3. In your clinical experience, what findings are predictive of severe illness in children?

The responses from Round 1 were summarised and feedback given to the panellists. In Rounds 2 and 3, participants were asked how strongly they agree with the inclusion of variables, considering the feedback from Round 1.

### Feature selection

Candidate features for data collection were identified by the researcher (implied elicitation) by combining the findings of the literature search and Delphi procedure. It was determined that features should be applicable to a wide range of clinical settings, particularly where specialised expertise and advanced investigations are not available. To that end the following eligibility criteria were set for variables:

1. No laboratory data were included (except point of care glucose testing).
2. Variables were such that a nurse or general practitioner could be expected to collect them in clinical consultations.
3. Variables were required to be relevant to the clinical services offered in the research site.

### Feature selection for data collection

Integrating the above, a list of features was selected for the data collection phase. Considering the results of Round 1 of the Delphi procedure, an approach that compromised between comprehensiveness, veracity, practicality and expected correlation with clinical outcome was chosen. Features of equivalent meaning were combined (accessory muscle use and grunting were combined as respiratory distress for example) or where a quantifiable candidate was available (such as in the case of cyanosis or peripheral oxygen saturation), the quantifiable metric was selected. For level of consciousness, both the AVPU scale (an ordinal score for consciousness—alert, response to verbal stimuli, response to pain, unresponsive) and a broader variable of altered level of consciousness were available (16). These features represent clinically detectable markers of specific organ dysfunctions. In view of the high prevalence of human immunodeficiency virus (HIV) and malnutrition in South Africa (17–19), the current status of HIV diagnosis and treatment together with anthropometry values were also included for collection as these were thought to be potentially informative features in this clinical setting.

## Results

### Literature search

The search procedure and results are summarised in Table 1.

**Table 1:** Literature search procedure.

Search terms	Title (“risk” or “predict*” or “score”) and title (“mortalit*” or “death”) and title (“child*” or “paed*” or “ped*”) not (neonat* or newborn*) not (“pugh”)
Database	Medline
Results	878
Eligible titles	275
Removed titles	23—articles prior to 2000 3—duplicates
Removed abstracts	51—ineligible 3—not available in English 41—no full-text available
Eligible full-texts	154
Setting	LMIC—89 High income—65

**Figure 2:**
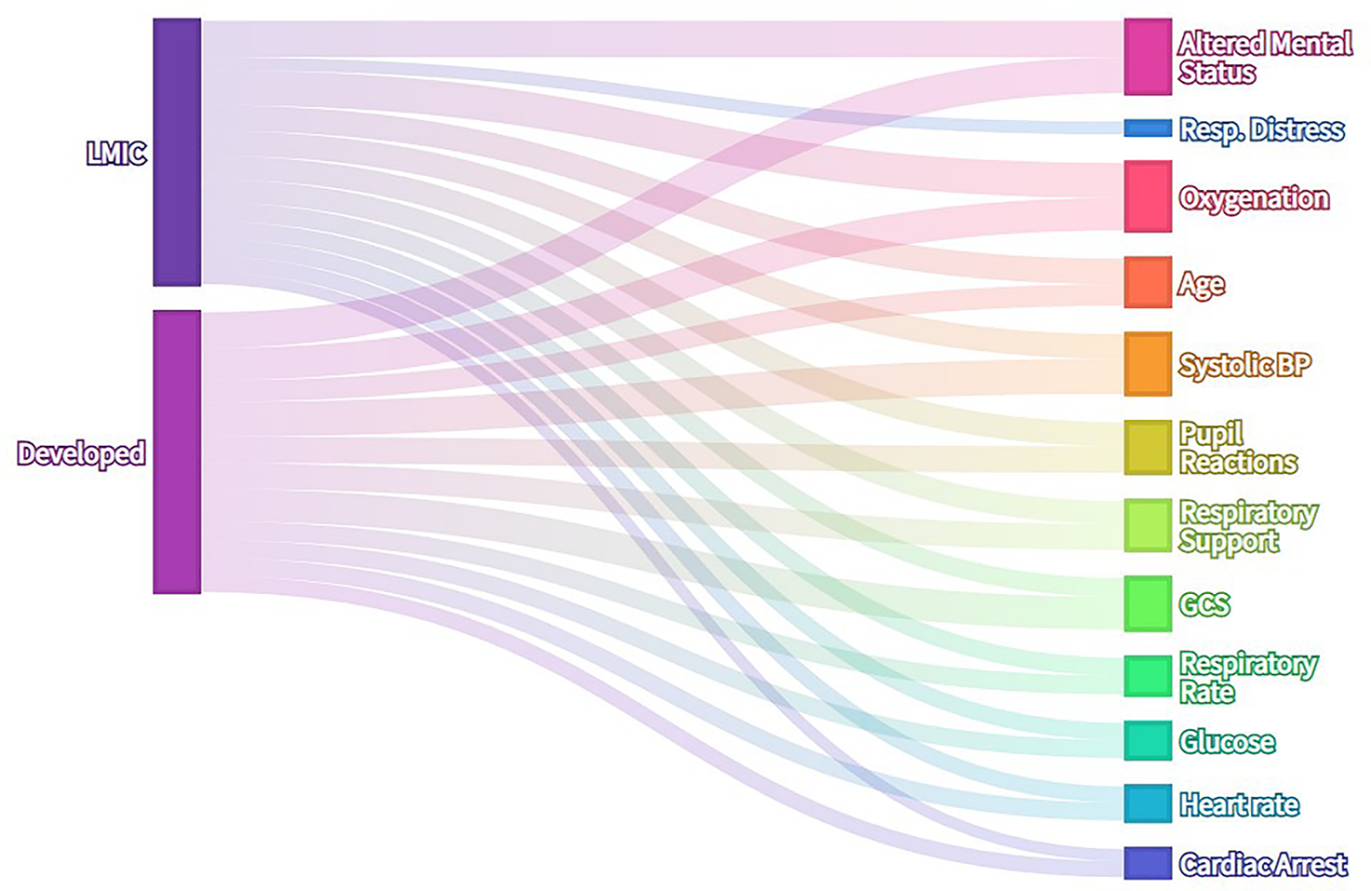
Alluvial diagram of literature search results—frequency with which clinical features are reported as risk factors for mortality in hospitalised children. Where features were present in existing predictive models, each feature was recorded as one instance. No laboratory data or special investigations except capillary blood glucose were included in analysis. Features present in more than 15% of publications are included in this visualization.

Features that have been found to be predictive of mortality in hospitalised children were recorded together with the number of participants in each study and pathology included in the study (for example, hospitalised children in general, pneumonia, malnutrition, burns etc.). Where predictive scoring systems or models were reported [such as the Paediatric Index of Mortality (20)], the eligible features included in the model were recorded as prognostic features. These results are summarised in Figure 2 and Supplementary Figure S1. in terms of the number of publications in which specific factors are reported to be predictive of mortality. A wide range of candidate features was identified by this process. In summary, the majority of features included were some measure of neurological, cardiovascular, respiratory or other organ dysfunction (jaundice, hypoglycaemia, hypothermia).

**Figure 3:**
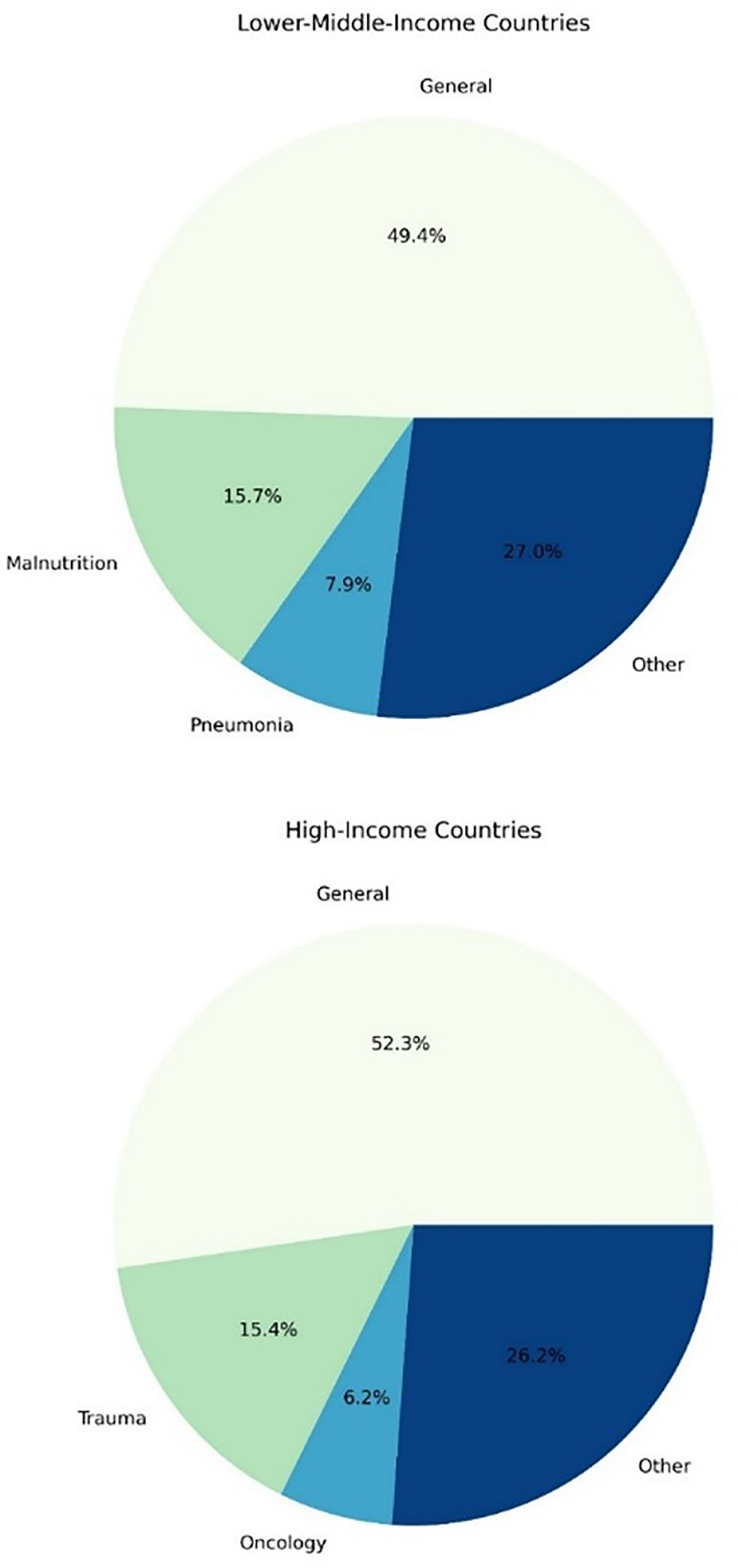
The frequency of pathological categories evaluated in studies reporting risk factors for mortality in hospitalised children.

The pathological categories in each group are presented in Figure 3. General populations of hospitalised children made up the majority of the literature, with malnutrition and pneumonia also being significant contributors in the LMIC group and trauma in the high-income group.

### Delphi

The response rate for Round 1 of the Delphi procedure was 91.67%. Free text responses were analysed thematically and collated. The responses are summarised in Table 2.

**Table 2:** Expert responses from round 1 of delphi procedure.

Question 1	Question 2	Question 3
What are important characteristics of a variable for inclusion in a study designed to predict severe illness in children in a resource limited setting?	Should completeness or practicality of data collection completeness be given priority in data collection for this study?	In your clinical experience, what findings are predictive of severe illness in children?
***Theme 1: Veracity: (9 respondents, 81.88%)*** Well defined/standardized/repeatable/objectivity (8 respondents, 72.72%) Negative predictive value/specificity (2 respondents, 18.18%) Positive predictive value (1 respondent, 9.09%) Independent of application of therapy (1 respondent, 9.09%) ***Theme 2: Integratedness and comprehensiveness (6 respondents, 54.54%)*** Part of an early warning system (1 respondent, 9.09%) Can be tracked over time (2 respondents, 18.18%) Context specific (1 respondent, 9.09%) Individualised (1 respondent, 9.09%) Features linked to each other (1 respondent, 9.09%) Include history of illness (1 respondent, 9.09%) ***Theme 3: Practicality of use (7 respondents, 63.63%)*** Easy to elicit/easy to use (6 respondents, 54.54%) Should not require interpretation prior to collection (1 respondent, 9.09%) Should not require expensive equipment/cheap to use (2 respondent, 18.18%) Part of routinely collected data (2 respondents, 18.18%) ***Theme 4: Correlation with severity of illness (4 respondents, 40%)*** Tracks changes in clinical condition (2 respondents, 18.18%) Indicative of physiological abnormality (1 respondent, 9.09%) Emphasises danger signs (1 respondent, 9.09%)	***Completeness (2 respondents, 18.18%*)** ***Practicality (2 respondents, 18.18%)*** ***A compromise between practicality and completeness (7 respondents 63.63%***	***Cardiovascular signs (8 respondents, 72.72%)*** Pulse rate (7 respondents, 63.63%) Tachycardia (7 respondents, 63.63%) Bradycardia (2 respondents, 18.18%) Poor peripheral perfusion (6 respondents, 54.54%) Prolonged capillary refill time (2 respondents, 18.18%) Weak pulses (3 respondents, 27.27% Cold skin (1 respondent, 9.09%) Mottled skin (1 respondent, 9.09%) Hypotension—5 respondents, 45.45% *Hypoxia or hypoxaemia (6 respondents, 54.54%)* Cyanosis (3 respondents, 27.27%) Low SPO_2_ (5 respondents, 45.45%) ***Respiratory signs 8 respondents, 72.72%*** Abnormal work of breathing (7 respondents, 63.63%) Tachypnoea (5 respondents, 45.45%) Bradypnoea (1 respondent, 9.09%) Chest indrawing (1 respondent 9.09%) Respiratory distress (3 respondents, 27.27%) Grunting (1 respondent 9.09%) Accessory muscle usage (2 respondents, 18.18%) ***Neurological signs (6 respondents, 54.54%)*** Altered activity levels (6 respondents, 54.54%) Lethargy (3 respondents, 30%) Altered level of consciousness (4 respondents, 36.36%) Neck stiffness (1 respondent, 9.09%) Hypotonia (1 respondent, 9.09%) Acute onset neurological signs (1 respondent, 9.09%) *Abnormal blood glucose (6 respondents, 54.54%)* Hypoglycaemia (5 respondents, 45.45%) Hyperglycaemia (1 respondent, 9.09%) ***Gastrointestinal signs (1 respondent, 9.09%*** Severe vomiting (1 respondent, 9.09%) Diarrhoea (1 respondent, 9.09%) ***Oliguria (2 respondents, 18.18%)*** ***Malnutrition (2 respondents, 18.18%)*** ***General Findings*** Not taking feeds or oral fluids (3 respondents, 27.27%) Underlying chronic disease (1 respondent, 9.09%) Age (2 respondents, 18.18%) Abnormal temperature (3 respondents, 27.27%) Fever (3 respondents, 27.27%) Hypothermia (1 respondent, 9.09%) Vital Signs (1 respondent, 9.09%) Organ dysfunction (1 respondent, 9.09%)

**Figure 4:**
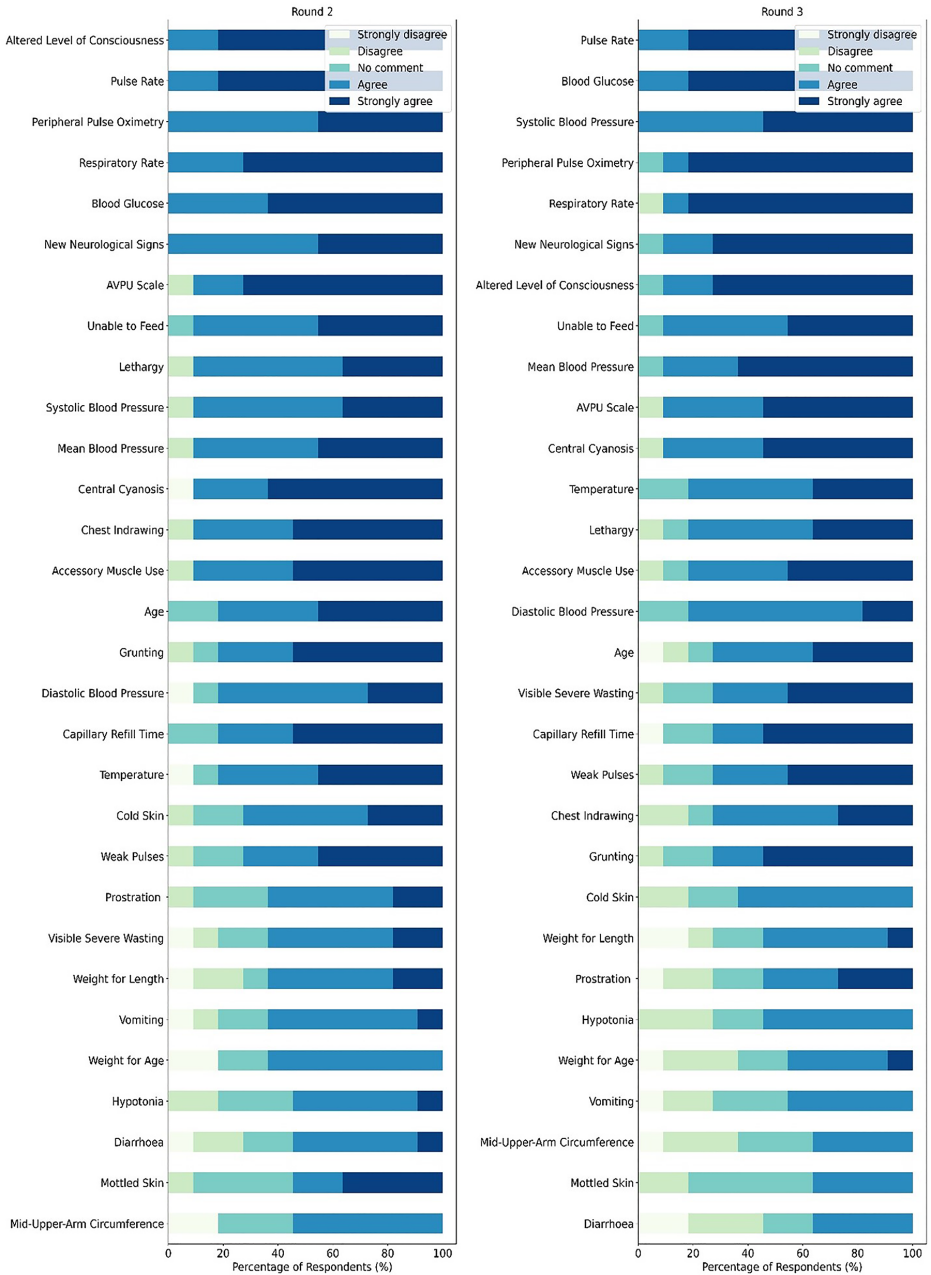
Stacked bar plot of agreement on features associated with severe illness in children.

These findings were provided to participants in writing at the start of Round 2. The response rate for both Rounds 2 and 3 was 91.67%. The results of Rounds 2 and 3 are presented in Supplementary Table S1 and Figure 4.

The list of features included 21 features which were collected is presented in Table 3.

**Table 3:** Selected features for data collection.

Continuous features	Categorical features
Age (months)	**Deep breathing** No/Yes
Respiratory rate (breaths per minute)	**Weak pulse** No/Yes
Peripheral saturation of oxygen (%)	**Level of consciousness** Alert/Prostrate/Coma
Pulse (beats per minute)	**AVPU scale** Alert/Verbal/Pain/Unresponsive
Systolic blood pressure (mmHg)	**Unable to feed** No/Yes
Diastolic blood pressure (mmHg)	**Respiratory distress** No/Yes
Capillary refill time (sec)	**Jaundice** No/Yes
Weight (kg)	**Seizures** No/Yes
Height (cm)	**Respiratory support** Room Air/Nasal Cannula/Intubated
Temperature (°C)	**HIV Infection** Unexposed Exposed, uninfected Infected on treatment <3 months Infected on treatment ≥3 months Infected, untreated Unknown
Glucose (mmol/L)	**Outcome** Died/PICU Combined outcome

## Discussion

In this study we have described the design of a dataset for a machine learning model designed to detect critically ill children in a hospital setting. This model aims to address the contribution of delays in identification, resuscitation and referral to avoidable severity of illness and mortality in South African Children (3). To achieve this, a documented process of literature search and expert consensus was undertaken to identify, and engineer candidate features and set operational priorities for the intended application. The taxonomy provided by Kerrigan et al. (7) provided a useful framework to represent the elicitation process. In this study, domain knowledge from experts, a literature search and the researcher were integrated to achieve the goals of problem specification and feature selection prior to data collection, feature engineering and pre-processing.

A documented and transparent Delphi procedure and literature search provide a more careful and rigorous approach to the elicitation of domain knowledge over unstructured or *ad-hoc* approaches to decision making (13, 15). This makes a useful contribution to the description of methods for domain knowledge elicitation in applied medical machine learning.

The elicitation of human domain knowledge is not directly addressed in the publications on machine learning models for PICU mortality prediction published by Aczon et al. and Kim et al. (10, 11). Goto et al. report the use of *a priori* knowledge in their model for prediction of clinical outcomes for children undergoing emergency department triage, but do not elaborate further (9).

The Transparent Reporting of multivariable prediction model of Individual Prognosis or Diagnosis (TRIPOD) statement provides a rigorous guideline for the publication of predictive models (21). The inclusion of robust descriptions of the elicitation of domain knowledge would further strengthen the ability of such guidelines to promote rigour and transparency in the reporting of predictive models and other applied machine learning models in medical research.

## Conclusion

The integration of domain knowledge is an important aspect of applied machine learning in medicine. The documentation of this process for eliciting such knowledge promotes the transparent and rigorous reporting of machine learning models and should be seen as an important methodological aspect of such research. In this study we have described and documented the use of a Delphi procedure and literature search as part of a method for problem specification, data set development and feature engineering processes in model development. These models are intended for the identification of critically ill children in South Africa. This process aimed to ensure that suitable features are made available during the development, training and testing phases in order to develop a usable, context-appropriate predictive model that addresses the clinical problem of delays in the recognition, resuscitation and referral of children with severe illness.

## Data Availability

The raw data supporting the conclusions of this article will be made available by the authors, without undue reservation.
