# Supplementary material for: Elicitation of domain knowledge for a machine learning model for paediatric critical illness in South Africa
Source: Front Pediatr. 2023 Feb 21;11:1005579. doi: 10.3389/fped.2023.1005579 (PMC9989015; doi:10.3389/fped.2023.1005579)
Supplement: Supplementary file 1 [file Table1.docx]

|  | Median Round 2 | IQR Round 2 | Agreement Round 2 (%) | Median Round 3 | IQR Round 3 | Delta Score | Agreement Round 3(%) | Delta Agreement (%) |
| --- | --- | --- | --- | --- | --- | --- | --- | --- |
| Pulse Rate | 5.00 | 0.00 | 100.00 | 5.00 | 0.00 | 0.00 | 100.00 | 0.00 |
| Blood Glucose | 5.00 | 1.00 | 100.00 | 5.00 | 0.00 | 0.00 | 100.00 | 0.00 |
| Systolic Blood Pressure | 4.00 | 1.00 | 90.91 | 5.00 | 1.00 | 1.00 | 100.00 | 9.09 |
| Peripheral Pulse Oximetry | 4.00 | 1.00 | 100.00 | 5.00 | 0.00 | 1.00 | 90.91 | -9.09 |
| New Neurological Signs | 4.00 | 1.00 | 100.00 | 5.00 | 0.50 | 1.00 | 90.91 | -9.09 |
| Altered Level of Consciousness | 5.00 | 0.00 | 100.00 | 5.00 | 0.50 | 0.00 | 90.91 | -9.09 |
| Respiratory Rate | 5.00 | 0.50 | 100.00 | 5.00 | 0.00 | 0.00 | 90.91 | -9.09 |
| Unable to Feed | 4.00 | 1.00 | 90.91 | 4.00 | 1.00 | 0.00 | 90.91 | 0.00 |
| AVPU Scale | 5.00 | 0.50 | 90.91 | 5.00 | 1.00 | 0.00 | 90.91 | 0.00 |
| Central Cyanosis | 5.00 | 1.00 | 90.91 | 5.00 | 1.00 | 0.00 | 90.91 | 0.00 |
| Mean Blood Pressure | 4.00 | 1.00 | 90.91 | 5.00 | 1.00 | 1.00 | 90.91 | 0.00 |
| Diastolic Blood Pressure | 4.00 | 0.50 | 81.82 | 4.00 | 0.00 | 0.00 | 81.82 | 0.00 |
| Lethargy | 4.00 | 1.00 | 90.91 | 4.00 | 1.00 | 0.00 | 81.82 | -9.09 |
| Temperature | 4.00 | 1.00 | 81.82 | 4.00 | 1.00 | 0.00 | 81.82 | 0.00 |
| Accessory Muscle Use | 5.00 | 1.00 | 90.91 | 4.00 | 1.00 | -1.00 | 81.82 | -9.09 |
| Age | 4.00 | 1.00 | 81.82 | 4.00 | 1.50 | 0.00 | 72.73 | -9.09 |
| Capillary Refill Time | 5.00 | 1.00 | 81.82 | 5.00 | 1.50 | 0.00 | 72.73 | -9.09 |
| Grunting | 5.00 | 1.00 | 81.82 | 5.00 | 1.50 | 0.00 | 72.73 | -9.09 |
| Chest Indrawing | 5.00 | 1.00 | 90.91 | 4.00 | 1.00 | -1.00 | 72.73 | -18.18 |
| Visible Severe Wasting | 4.00 | 1.00 | 63.64 | 4.00 | 1.50 | 0.00 | 72.73 | 9.09 |
| Weak Pulses | 4.00 | 1.50 | 72.73 | 4.00 | 1.50 | 0.00 | 72.73 | 0.00 |
| Cold Skin | 4.00 | 1.00 | 72.73 | 4.00 | 1.00 | 0.00 | 63.64 | -9.09 |
| Weight for Length | 4.00 | 1.50 | 63.64 | 4.00 | 1.50 | 0.00 | 54.55 | -9.09 |
| Prostration | 4.00 | 1.00 | 63.64 | 4.00 | 2.00 | 0.00 | 54.55 | -9.09 |
| Hypotonia | 4.00 | 1.00 | 54.55 | 4.00 | 1.50 | 0.00 | 54.55 | 0.00 |
| Weight for Age | 4.00 | 1.00 | 63.64 | 3.00 | 2.00 | -1.00 | 45.45 | -18.18 |
| Vomiting | 4.00 | 1.00 | 63.64 | 3.00 | 1.50 | -1.00 | 45.45 | -18.18 |
| Diarrhoea | 4.00 | 1.50 | 54.55 | 3.00 | 2.00 | -1.00 | 36.36 | -18.18 |
| Mid-Upper-Arm Circumference | 4.00 | 1.00 | 54.55 | 3.00 | 2.00 | -1.00 | 36.36 | -18.18 |
| Mottled Skin | 4.00 | 2.00 | 54.55 | 3.00 | 1.00 | -1.00 | 36.36 | -18.18 |

**Supplementary Table 1: Responses from Rounds 2 and 3 of Delphi Procedure - IQR: Interquartile Range. Agreement was calculated by determining the percentage of responses of "agree" or "strongly agree".**
