# Supplementary figures and images for: Elicitation of domain knowledge for a machine learning model for paediatric critical illness in South Africa
Source: Front Pediatr. 2023 Feb 21;11:1005579. doi: 10.3389/fped.2023.1005579 (PMC9989015; doi:10.3389/fped.2023.1005579)

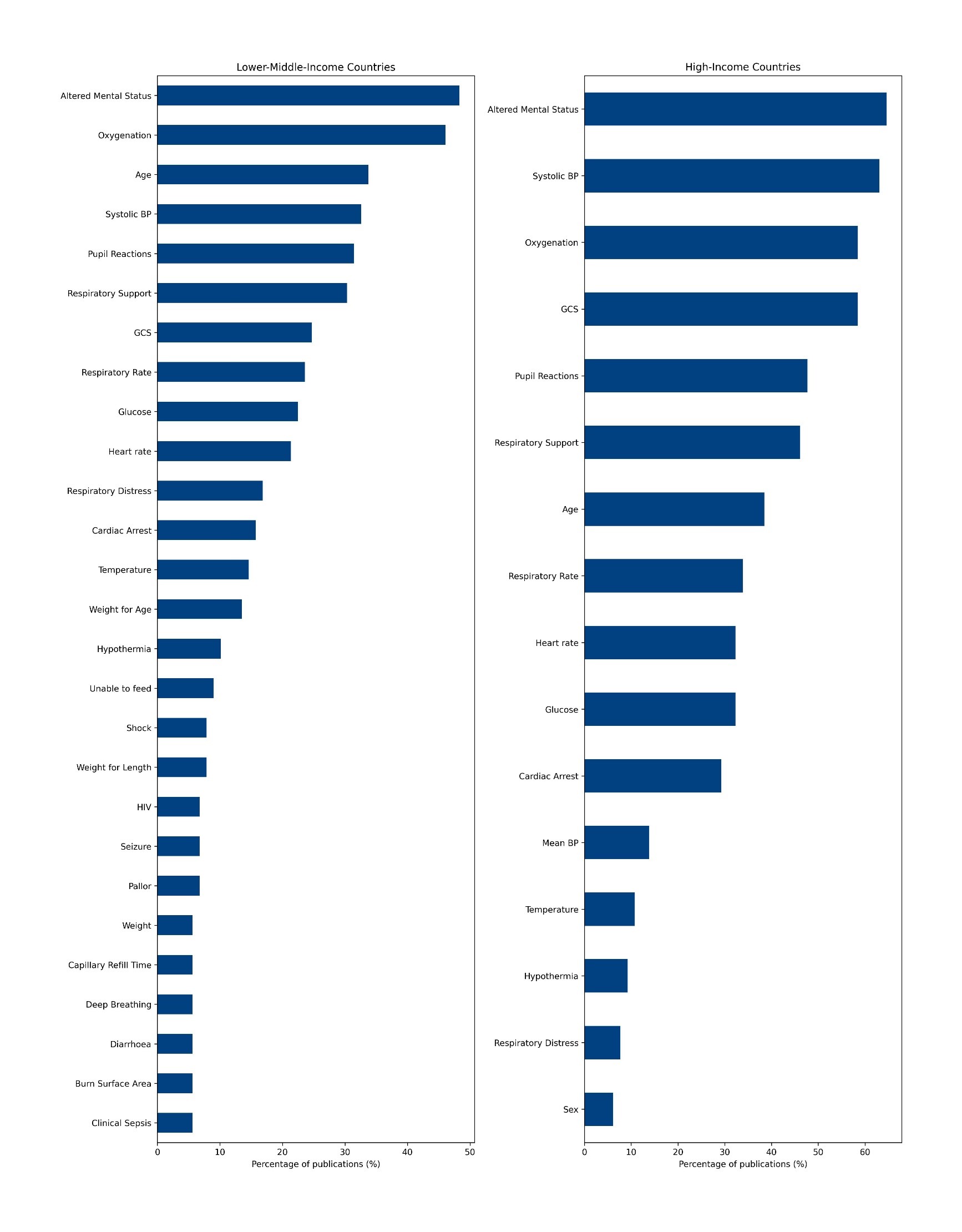

Supplement: Supplementary file 2 [file Image1.jpeg]
